# Characterization of Xyloglucanase TpXEG12a from *Talaromyces pinophilus*

**DOI:** 10.3390/ijms27010294

**Published:** 2025-12-27

**Authors:** Junhui Nie, Peng Li, Cheng Zhang, Jing Zeng, Siyuan Yue, Jianjun Guo, Dawei Xiong, Shuaiwen Zhang, Guochang Huang, Lin Yuan

**Affiliations:** Institute of Biomanufacturing, Jiangxi Academy of Sciences, Nanchang 330095, China

**Keywords:** xyloglucan, xyloglucanase, XEG, *Talaromyces pinophilus*, enzymatic properties

## Abstract

Xyloglucan, a key component of plant cell wall polysaccharides, plays a crucial role in cell wall structural remodeling and biomass recalcitrance. This study reports the discovery and biochemical characterization of a novel glycoside hydrolase family 12 (GH12) xyloglucanase, TpXEG12a, from the biomass-degrading fungus *Talaromyces pinophilus*. Recombinant TpXEG12a exhibited exceptional catalytic efficiency toward xyloglucan, with a specific activity of 2375 U/mg, significantly higher than the typical range reported for GH12 xyloglucanases. The enzyme displayed optimal activity at pH 4.0 and 57 °C, with high stability in acidic conditions (pH 4–8) and moderate thermal stability. TpXEG12a demonstrated strict substrate specificity for xyloglucan, with no detectable activity against cellulose-related substrates, and primarily generated characteristic xyloglucan oligosaccharides (XXXG, XLXG/XXLG, XLLG) upon hydrolysis. Structural analysis revealed that TpXEG12a exists as a stable homodimer in solution, which likely contributes to its catalytic efficiency. Notably, TpXEG12a synergistically enhanced glucose release when combined with cellulase in lignocellulosic biomass degradation. These findings establish TpXEG12a as a promising candidate for industrial applications in biomass conversion, textile processing, and functional oligosaccharide production.

## 1. Introduction

Xyloglucan serves as a key component of plant cell wall polysaccharides and plays a central role in cell wall structural remodeling and biomass recalcitrance [1,2]. This hemicellulose is particularly abundant in the primary cell walls of dicotyledons, where it cross-links cellulose microfibrils to form a dynamic cellulose-xyloglucan network, which is essential for regulating cell wall extensibility to support plant growth [1,3]. Microbial xyloglucanases specifically hydrolyze the β-1,4-glucan backbone of xyloglucan and play important roles in natural biomass degradation and industrial biorefining, such as in pretreatment steps for bioethanol production [4,5]. Among these, glycoside hydrolase family 12 (GH12) enzymes have attracted considerable interest due to their compact molecular structures and potential to degrade substrates under harsh conditions, such as high temperature and in the presence of crystalline regions [6,7]. However, current understanding of the biodiversity and biocatalytic adaptability of GH12 xyloglucanases, particularly regarding industrially relevant traits such as alkali tolerance and thermostability, remains incomplete. Therefore, continued extensive screening for novel and high-quality xyloglucanases from natural sources is warranted.

In biotechnological applications such as textile biostoning and bioethanol production, there is a strong industrial demand for efficient xyloglucanases that combine high stability (especially under high temperature and alkaline conditions), high substrate specificity (exclusively toward xyloglucan), and compatibility with process parameters (e.g., pH and temperature) [8]. Conventional cellulase preparations often suffer from insufficient endogenous xyloglucanase activity or concomitant significant cellulase activity, leading to undesirable hydrolysis of non-target substrates—for instance, compromising cotton fiber strength in denim stonewashing [8]. Consequently, researchers are committed to discovering novel xyloglucanases from unique sources that efficiently hydrolyze xyloglucan under high temperature and alkaline conditions while exhibiting minimal cellulase activity, thereby avoiding non-target cellulose degradation. Recent advances in genomics have accelerated the discovery of novel enzymes. A variety of microorganisms producing thermostable and alkali-tolerant xyloglucanases have been reported, including *Rhizomucor miehei*, *Aspergillus niveus*, *A. oryzae*, *Penicillium canescens*, *P. verruculosum*, *Debaryomyces hansenii*, *Thermomonospora* sp., *Ruminococcus flavefaciens*, and *Paenibacillus polymyxa* [7,9,10,11]. Although xyloglucanases in several CAZy families including GH5 and GH16 families have also been investigated, GH12 family alkali-tolerant and thermostable xyloglucanases that fully meet the stringent requirements of such industrial applications remain particularly scarce, limiting the development of high-performance industrial enzyme libraries [12,13,14].

To address the limited availability of high-performance xyloglucanases, this study reports the identification and biochemical characterization of a novel GH12 xyloglucanase, TpXEG12a, from *Talaromyces pinophilus*. Recombinant TpXEG12a exhibited high catalytic activity toward xyloglucan and strict substrate selectivity with minimal activity toward cellulose, highlighting its potential suitability for applications requiring selective hemicellulose hydrolysis. The enzyme was systematically characterized in terms of optimal pH and temperature, stability profiles, substrate specificity, and kinetic parameters, and its preliminary applicability was evaluated using soybean meal and corncob powder. These results expand the functional understanding of GH12 xyloglucanases and provide a basis for further engineering and application of TpXEG12a.

## 2. Results

### 2.1. Phylogenetic Analysis and Sequence Alignment of TpXEG12a

The protein encoded by TpXEG12a is annotated as a glycoside hydrolase family 12 (GH12) member in the genome of *Talaromyces pinophilus*. To investigate the evolutionary position of the *T. pinophilus*-derived xyloglucanase TpXEG12a within GH12, a phylogenetic tree was constructed using its amino acid sequence alongside sequences of xyloglucan-specific endo-β-1,4-glucanases from various fungi sourced from the NCBI database. The tree was built using the Neighbor-Joining (NJ) method, with branch lengths representing evolutionary distances between sequences.

**Figure 1:**
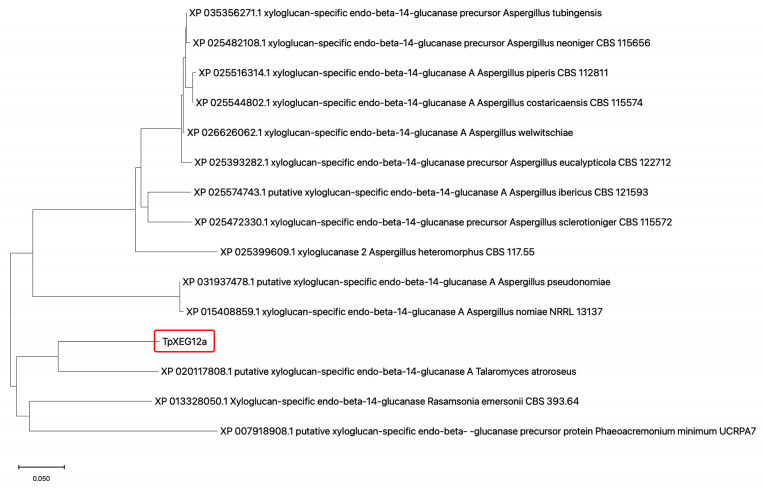
Phylogenetic analysis of the xyloglucanase TpXEG12a from T. pinophilus. A phylogenetic tree was constructed using the amino acid sequences of TpXEG12a and GH12 family xyloglucanases from various fungal genera, including Talaromyces, Aspergillus, Rasamsonia, and Phaeoacremonium. Sequence alignment was performed with ClustalW, and the tree was generated in MEGA 11 using the Neighbor-Joining (NJ) method with 1000 bootstrap replications to assess branch reliability [15]. Bootstrap values are indicated at the nodes, and branch lengths represent evolutionary distances [16,17].

The results (Figure 1) showed that TpXEG12a clustered with a putative xyloglucanase from Talaromyces atroroseus (XP_020117808.1), forming a distinct clade, which indicates a close evolutionary relationship between them and confirms their placement within the Talaromyces genus. This suggests that TpXEG12a may have retained characteristic Talaromyces structural domains while acquiring subtle sequence variations distinguishing it from other fungal GH12 xyloglucanases. In contrast, xyloglucanase sequences from the Aspergillus genus (e.g., from *A. nomiae*, *A. neoniger*, *A. costaricaensis*) formed several separate subclades, indicating significant interspecific diversification of GH12 xyloglucanases within this genus. The relatively long branch distance between the clade containing TpXEG12a and the Aspergillus sequence clusters suggests potential structural and functional divergence. Furthermore, TpXEG12a was distantly related to the xyloglucanase from Rasamsonia emersonii (XP_013328050.1) and the sequence from Phaeoacremonium minimum (XP_007918908.1), indicating that TpXEG12a belongs to a specific evolutionary lineage of fungal endo-xyloglucanases within GH12, rather than to broad-specificity endoglucanases.

**Figure 2:**
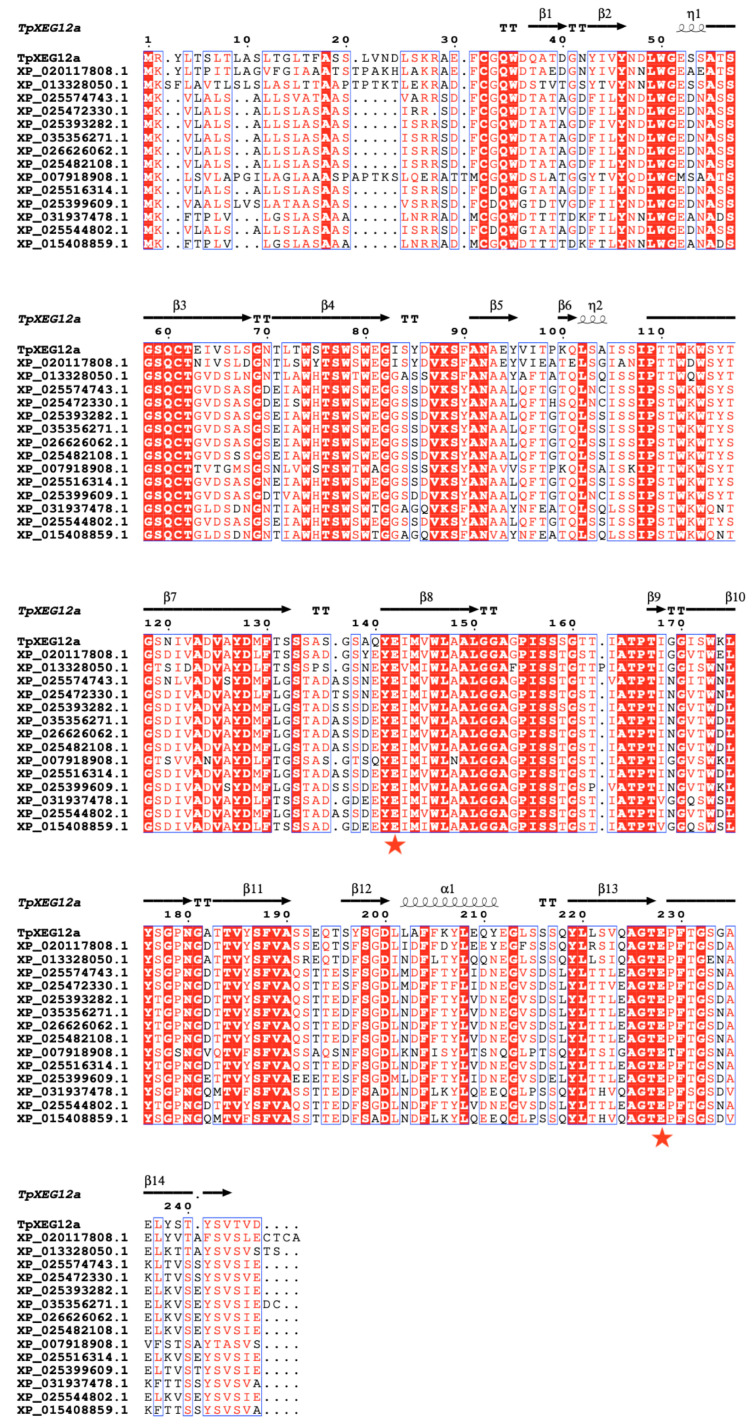
Multiple sequence alignment of TpXEG12a with homologous GH12 family xyloglucanases. Multiple sequence alignment of TpXEG12a and GH12 family xyloglucanases from various fungal sources was performed using Clustal Omega 1.2 and visualized with ESPript 3.0. Fully conserved residues are highlighted with a red background, while partially conserved residues are shown in red letters. Black arrows indicate β-strands (β1–β14), helical symbols represent α-helices and η-helices, and “TT” denotes turn regions. Red pentagrams mark the two conserved catalytic Glu residues, whose positions correspond to the typical catalytic motif of GH12 enzymes.

Multiple sequence alignment (Figure 2) revealed that TpXEG12a retains the characteristic β-jelly-roll fold and secondary structure elements typical of GH12, comprising 14 β-strands (β1–β14) and one α-helix. An N-terminal hydrophobic signal peptide was identified, consistent with TpXEG12a being a secreted extracellular enzyme. The catalytic center of TpXEG12a was highly conserved compared to other fungal xyloglucanases, with the two catalytic acidic residues entirely conserved, suggesting its catalytic mechanism is consistent with that of typical GH12 endo-β-1,4-glucanases. The alignment also showed that TpXEG12a possesses several conserved aromatic residues surrounding the substrate-binding cleft, which are potentially involved in substrate recognition and stacking. Notably, amino acid substitutions and short indels were observed in specific loop regions (particularly between β7–β8 and β12–β13), which might influence substrate specificity or thermostability. These features are consistent with the phylogenetic analysis, indicating that while TpXEG12a maintains the typical GH12 structural fold and catalytic mode, it has acquired unique sequence characteristics derived from the *Talaromyces* genus, providing a molecular basis for its potential distinct enzymatic properties.

### 2.2. Expression and Purification of TpXEG12a

**Figure 3:**
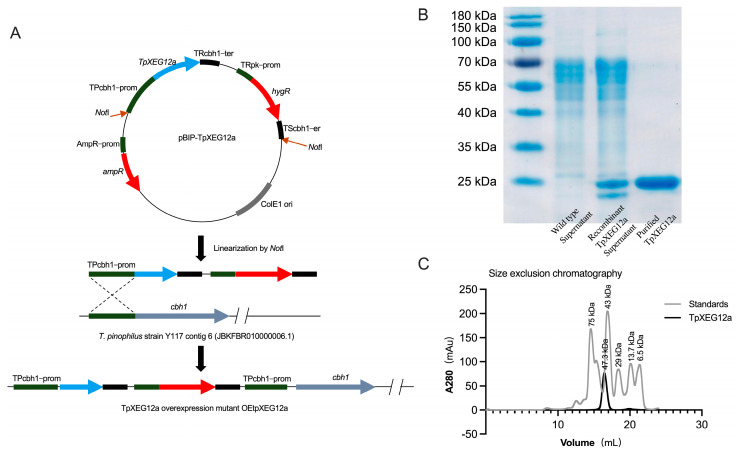
Construction of the overexpression vector, protein purification, and molecular mass analysis of TpXEG12a in *T. pinophilus*. (**A**) Schematic representation of the construction of the TpXEG12a overexpression vector pBIP-TpXEG12a and its genomic integration strategy in *T. pinophilus*. The TpXEG12a gene was expressed under the control of the endogenous cbh1 promoter (TPcbh1-prom) from *T. pinophilus*. After Not I linearization, the vector was integrated into the cbh1 locus via homologous recombination, yielding the overexpression mutant strain OEtpXEG12a. (**B**) SDS-PAGE analysis of purified TpXEG12a. The purified recombinant TpXEG12a showed a single distinct band at approximately 25 kDa on 12% SDS-PAGE. (**C**) Gel filtration chromatography analysis of TpXEG12a. Based on calibration with molecular mass standards (gray line), the major elution peak of TpXEG12a (black line) corresponded to an apparent molecular mass of approximately 47.3 kDa.

To obtain sufficient amounts of TpXEG12a protein, *T. pinophilus* was genetically engineered for homologous overexpression (Figure 3A). The TpXEG12a gene was placed under the control of the strong constitutive promoter TPcbh1 and inserted into the expression vector pBIP-TpXEG12a. After linearization with NotI, the construct was integrated into the host genome at the cbh1 locus via homologous recombination, yielding the overexpression mutant OEtpXEG12a. This strategy ensured stable expression of TpXEG12a within *T. pinophilus* while minimizing potential disruption to non-target genomic regions beyond the major cellulase gene locus.

As shown in Figure 3B, the recombinant TpXEG12a protein purified by Ni^2+^ affinity chromatography appeared as a single band of approximately 25 kDa on SDS–PAGE, consistent with its predicted molecular weight of 23.3 kDa, indicating high purity of the recombinant enzyme. The absence of detectable contaminant bands further confirmed that TpXEG12a was efficiently expressed in a soluble form in the host.

To further investigate the oligomeric state of TpXEG12a, size-exclusion chromatography (SEC) was performed for molecular mass determination (Figure 3C). TpXEG12a eluted as a single symmetrical peak corresponding to an apparent molecular mass of approximately 47.3 kDa, nearly twice that estimated from SDS–PAGE, suggesting that the enzyme predominantly exists as a homodimer in solution.

### 2.3. Signal Peptide of TpXEG12a

**Figure 4:**
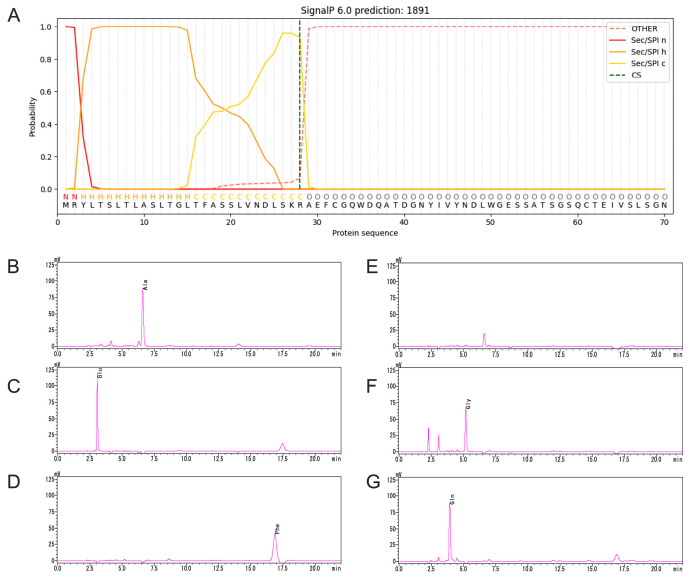
Identification of the signal peptide of TpXEG12a. (**A**) SignalP 6.0 prediction of the N-terminal signal peptide of TpXEG12a. The model indicates a typical fungal Sec/SPI-type signal peptide spanning residues 1–28, consisting of a positively charged N-region, a hydrophobic h-region, and a short C-region adjacent to the predicted cleavage site (vertical dashed line). (**B**–**G**) N-terminal sequencing of purified TpXEG12a by Edman degradation. The chromatograms show the sequential identification of the first six residues (A–E–F–X–G–Q) of the mature secreted enzyme, matching the amino acid sequence immediately following the predicted cleavage site before Ala^29^.

To identify the signal peptide region of TpXEG12a, the amino acid sequence of TpXEG12a was analyzed using SignalP 6.0. The prediction revealed that the N-terminus of TpXEG12a contains a typical 28-amino-acid signal peptide with the sequence MRYLTSLTLASLTGLTFASSLVNDLSKR (Figure 4A). This region is rich in hydrophobic residues and exhibits the characteristic architecture of fungal signal peptides, including a positively charged N-region, a hydrophobic h-region, and a short, neutral C-region near the cleavage site. These features are consistent with those commonly observed in fungal secretory proteins.

To experimentally validate the signal peptide prediction, N-terminal amino acid sequencing of the purified TpXEG12a protein was performed using the Edman degradation method. The analyzed protein corresponded to the mature enzyme secreted into the culture supernatant after signal peptide cleavage. The sequencing results showed that the first six residues of the mature TpXEG12a were A–E–F–X–G–Q (Figure 4B–G), which precisely matched the expected residues following the predicted cleavage site. This confirmed that the signal peptide of TpXEG12a comprises the first 28 amino acids, with the cleavage occurring before Ala^29^. Therefore, TpXEG12a is identified as a typical secreted xyloglucanase from *T. pinophilus*.

### 2.4. Biochemical Characterization of TpXEG12a

**Figure 5:**
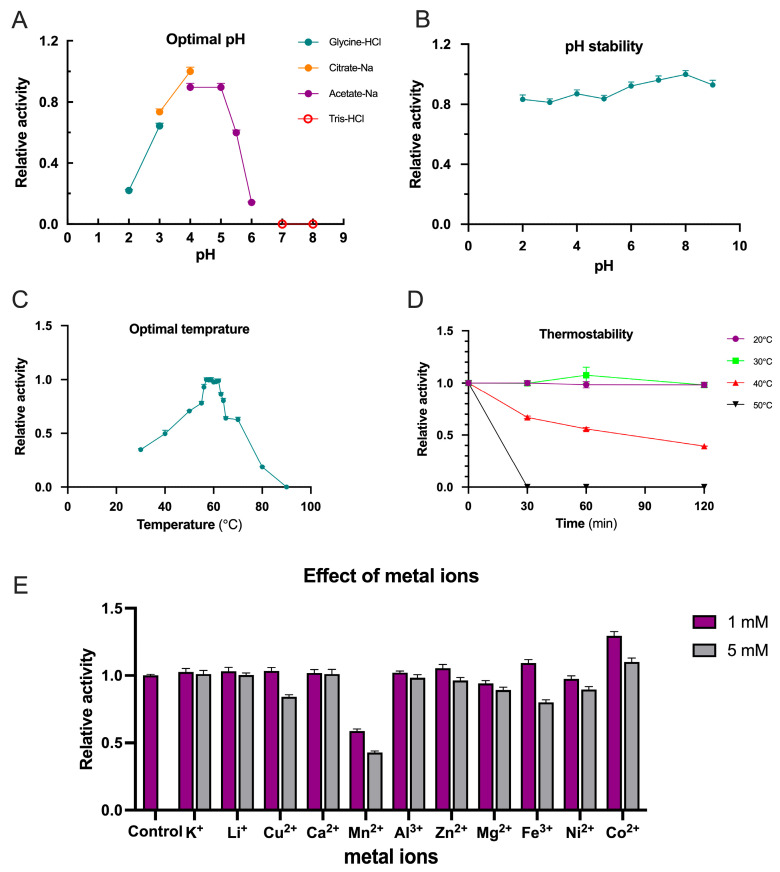
Biochemical characterization of TpXEG12a. (**A**) Effect of pH on TpXEG12a activity in different buffer systems (Glycine–HCl, Citrate–Na, Acetate–Na, and Tris–HCl, each 50 mM). (**B**) pH stability of TpXEG12a after incubation at various pH values (4.0–8.0) for 1 h at room temperature. (**C**) Temperature dependence of TpXEG12a activity. (**D**) Thermostability analysis of TpXEG12a after incubation at 20 °C, 30 °C, 40 °C, and 50 °C for up to 120 min. (**E**) Effects of different metal ions (1 and 5 mM) on TpXEG12a activity.

The enzymatic activity of TpXEG12a toward xyloglucan substrates was assessed under various pH conditions (1% substrate concentration) at 50 °C. As shown in Figure 5A, TpXEG12a exhibited high catalytic activity over a pH range of 3.0–5.5, with an optimum pH around 4.0. The activity decreased sharply when the pH dropped below 3 or exceeded 6, and the enzyme was completely inactivated at pH 7. In terms of pH stability, TpXEG12a remained highly stable after incubation at different pH values for 60 min, showing negligible loss of activity (Figure 5B).

The optimal temperature for TpXEG12a activity was determined at pH 4.0. As illustrated in Figure 5C, TpXEG12a showed maximum activity at 57 °C, followed by a rapid decline beyond 62 °C. Approximately 20% of the activity was retained at 80 °C, while the enzyme was completely inactivated at 90 °C. The thermostability of TpXEG12a was further evaluated at different incubation temperatures (Figure 5D). The enzyme retained nearly 100% activity after 120 min at 30 °C. At 40 °C, approximately 30% activity loss was observed after 30 min and 60% loss after 120 min. However, at 50 °C, the activity was completely lost within 30 min, indicating moderate thermal stability under acidic conditions.

To examine the influence of metal ions on TpXEG12a activity, various ions were added to the standard reaction mixture at final concentrations of 1 mM and 5 mM (Figure 5E). The results indicated that 5 mM Cu^2+^ inhibited the enzyme by approximately 15%, while Mn^2+^ caused ~40% and ~60% inhibition at 1 mM and 5 mM, respectively. Similarly, 5 mM Fe^3+^ led to a ~20% reduction in activity. In contrast, 1 mM Co^2+^ enhanced TpXEG12a activity by approximately 30%, whereas the stimulatory effect was less pronounced at 5 mM. Other tested metal ions, including Li^+^, Ca^2+^, Zn^2+^, Mg^2+^, Ni^2+^, and Al^3+^, had no significant effect on the enzyme’s catalytic performance.

### 2.5. Enzymatic Kinetics and Hydrolysis Product Analysis of TpXEG12a

**Figure 6:**
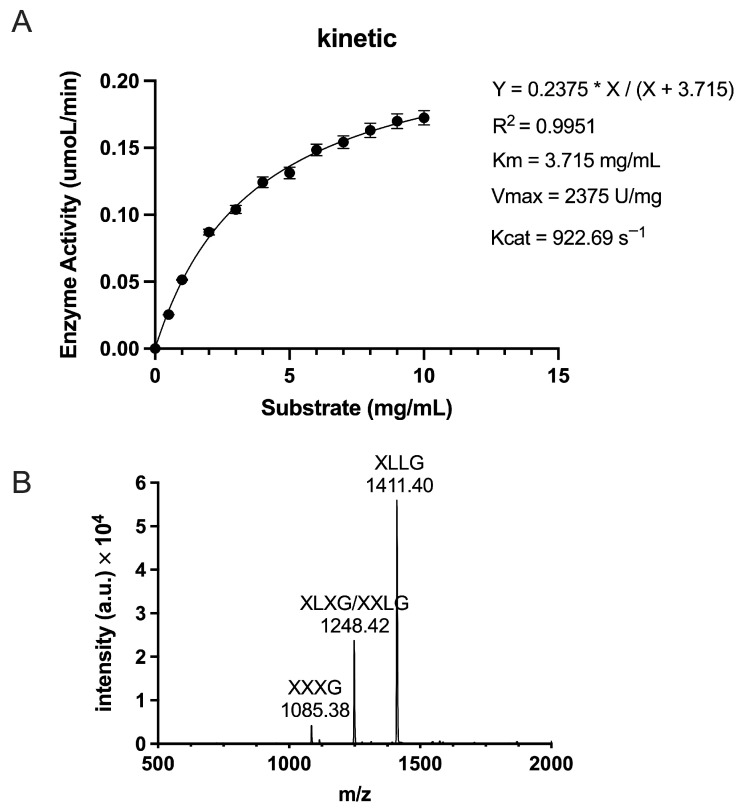
Enzymatic kinetics and hydrolysis product analysis of TpXEG12a. (**A**) Enzyme activity of TpXEG12a toward tamarind xyloglucan at different substrate concentrations (0.5–10 mg/mL). The kinetic data were fitted using the Michaelis–Menten equation Y = 0.2375 × X/(X + 3.715), yielding kinetic parameters of Km = 3.715 mg/mL, Vmax = 2375 U/mg, and Kcat = 922.69 s^−1^. (**B**) MALDI-TOF MS spectrum of hydrolysis products from tamarind xyloglucan degraded by TpXEG12a. The major oligosaccharides detected were XXXG (*m*/*z* = 1085.38), XLXG/XXLG (*m*/*z* = 1248.42), and XLLG (*m*/*z* = 1411.40).

To evaluate the catalytic performance of TpXEG12a toward xyloglucan, enzyme activity assays were performed under the optimal reaction conditions described in the Materials and Methods. Xyloglucan concentrations ranging from 0.5 to 10.0 mg/mL were used, and initial reaction rates were plotted as a function of substrate concentration to generate a kinetic curve. The data were fitted by nonlinear regression using the Michaelis–Menten model (Figure 6A). TpXEG12a exhibited a Km value of 3.715 mg/mL, indicating a high affinity for xyloglucan, together with a Vmax of 2375 U/mg and a turnover number (kcat) of 922.69 s^−1^, resulting in a catalytic efficiency (kcat/Km) of 248.37 s^−1^·mg^−1^·mL. A comparison with previously reported GH12 xyloglucanases (Table S1) demonstrates that TpXEG12a possesses an unusually high catalytic efficiency and strict substrate specificity. Collectively, these results indicate that TpXEG12a combines efficient substrate binding with rapid catalytic turnover, underscoring its potential for applications requiring selective and efficient xyloglucan degradation.

To elucidate the mode of action and substrate recognition of TpXEG12a, the enzymatic hydrolysis products of tamarind xyloglucan were analyzed using Matrix-Assisted Laser Desorption/Ionization Time-of-Flight Mass Spectrometry (MALDI-TOF MS) (Figure 6B). The major hydrolysis products were identified as XXXG (*m*/*z* = 1085.38), XLXG/XXLG (*m*/*z* = 1248.42), and XLLG (*m*/*z* = 1411.40), which are typical partial hydrolysis fragments of xyloglucan [18]. These results indicate that TpXEG12a specifically cleaves the β-1,4-glucosidic linkages within the xyloglucan backbone. No glucose, cellobiose, or other low-molecular-weight sugars were detected, confirming that TpXEG12a displays high substrate specificity with negligible activity toward cellulose-related polysaccharides.

### 2.6. Substrate Specificity

The substrate specificity of the recombinant enzyme TpXEG12a was assessed using a panel of potential substrates, including carboxymethyl cellulose (CMC), filter paper, xylan, and β-glucan. No hydrolytic activity against any of these substrates was detected, indicating that TpXEG12a exhibits high substrate specificity.

### 2.7. Structural Prediction, Molecular Docking, and Identification of Key Catalytic Residues of TpXEG12a

**Figure 7:**
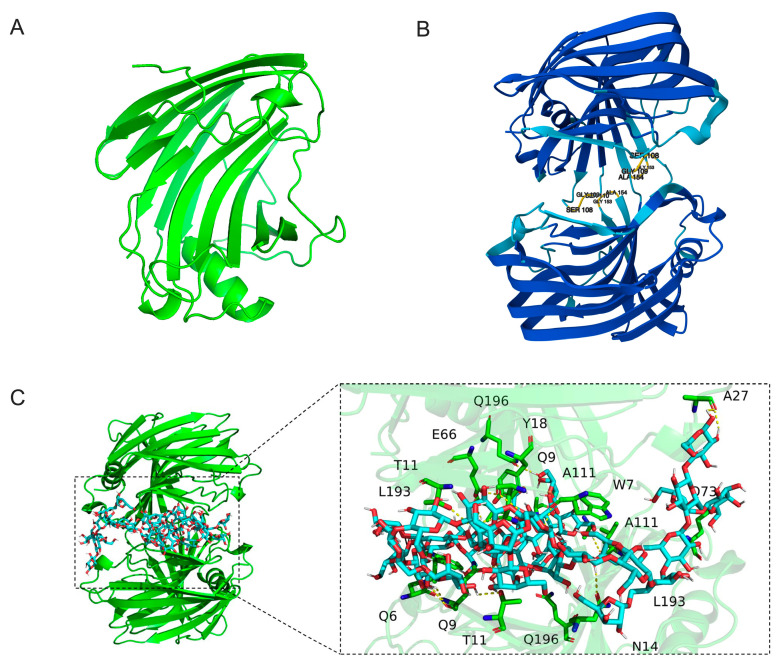
Structural prediction and molecular docking analysis of TpXEG12a. (**A**) Predicted monomeric structure of TpXEG12a generated by AlphaFold 3, showing the typical GH12 family β-jelly roll fold composed of two antiparallel β-sheets and a short α-helix. (**B**) Dimeric structure model of TpXEG12a, in which two monomers form a stable complex through a hydrophobic interface. (**C**) Molecular docking of xyloglucan with TpXEG12a. Close-up view of the binding mode between TpXEG12a and the xyloglucan substrate. The xyloglucan ligand is shown in a stick representation with increased thickness, and the carbon atoms of the substrate are colored distinctly to differentiate it from the surrounding amino acid residues. The docked substrate corresponds to an XXXG-type xyloglucan oligomer composed of a β-(1 → 4)-linked glucan backbone substituted with α-D-xylopyranosyl residues.

The AlphaFold 3 prediction revealed that TpXEG12a adopts a canonical structure characteristic of glycoside hydrolase family 12 (GH12) enzymes. The overall architecture displays a compact β-jelly roll fold, consisting of two antiparallel β-sheets forming a tightly packed β-sandwich structure, interspersed with several short α-helices and loop regions (Figure 7A). This highly conserved topology is a hallmark of GH12 enzymes and is thought to contribute to the remarkable conformational stability of these enzymes under extreme conditions, such as elevated temperatures or organic solvent environments [19,20].

In the predicted dimeric model (Figure 7B), two TpXEG12a monomers are associated through a network of hydrophobic interactions involving residues such as L193, A111, and Q196, located along the edges of the β-sheets. The resulting dimer interface is compact, with no evident steric clashes, supporting the notion that TpXEG12a likely exists as a functional homodimer in its native state. Such dimerization is proposed to enhance the structural stability of the substrate-binding cleft and the resilience of the catalytic core, a phenomenon also reported in certain thermostable fungal GH12 enzymes [21].

The molecular docking analysis (Figure 7C) further elucidated the interaction pattern between TpXEG12a and its xyloglucan substrate. The substrate chain was observed to extend along the substrate-binding groove, stabilized primarily by hydrogen bonding and van der Waals interactions. Key residues involved in substrate recognition include N14, T11, Y18, A27, E66, Q73, A111, L193, and Q196, which interact with the glucose backbone or xylose side chains of xyloglucan. The electrostatic surface potential of the binding groove exhibits alternating positive and negative patches, which favor stable interactions with the hydroxyl-rich polysaccharide substrate. Collectively, these structural insights suggest that TpXEG12a possesses a robust substrate recognition mechanism and an optimized catalytic environment, which likely underpin its high catalytic efficiency toward xyloglucan.

### 2.8. Synergistic Action Between TpXEG12a and Cellulase

**Figure 8:**
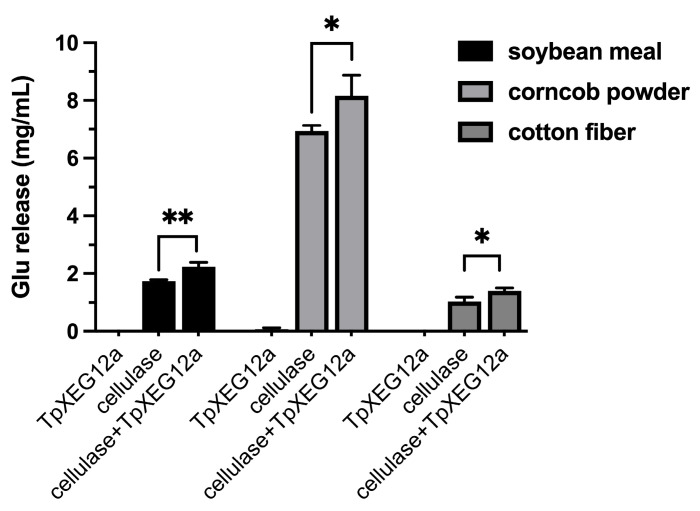
Synergistic saccharification of lignocellulosic substrates by TpXEG12a and cellulase. Glucose release from three lignocellulosic substrates—soybean meal, corncob powder, and cotton fiber—following treatment with TpXEG12a alone, cellulase alone, or a combination of both enzymes. Data are presented as mean ± SD (*n* = 3). Asterisks indicate statistically significant differences compared with cellulase alone (* *p* < 0.05, ** *p* < 0.01).

When applied individually to complex lignocellulosic substrates—including soybean meal, corncob powder, and cotton fiber—the recombinant xyloglucanase TpXEG12a released only trace amounts of glucose. In contrast, treatment with a commercial cellulase preparation alone yielded a measurable quantity of glucose, consistent with its known capacity to hydrolyze cellulose components. Notably, co-application of TpXEG12a with cellulase resulted in varying but consistently enhanced glucose release across all tested substrates. The observed increase in glucose yields significantly exceeded the sum of the amounts released by each enzyme acting alone, indicating a pronounced synergistic interaction between TpXEG12a and cellulase (Figure 8). These findings suggest that TpXEG12a efficiently cleaves xyloglucan cross-links embedded within the hemicellulose–cellulose matrix, thereby improving cellulase accessibility to cellulose and facilitating a more efficient saccharification process.

## 3. Discussion

In this study, a novel GH12 family xyloglucanase, TpXEG12a, derived from *T. pinophilus*, was systematically identified and characterized. The enzyme exhibits outstanding catalytic performance, distinct structural features, and promising industrial potential. TpXEG12a showed remarkably high catalytic efficiency toward tamarind xyloglucan, with Vmax and kcat values of 2375 U mg^−1^ and 922.69 s^−1^, respectively—significantly higher than most previously reported GH12 xyloglucanases [19,21,22]. These results indicate that TpXEG12a is among the most catalytically efficient GH12 xyloglucanases identified to date, providing valuable insight into the structural and functional diversity of fungal GH12 enzymes.

The superior catalytic performance of TpXEG12a may arise partly from its structural conformation and substrate interaction pattern. Predicted 3D modeling revealed a typical GH12 β-jelly roll (β-sandwich) fold, consisting of two antiparallel β-sheets—a feature that contributes to molecular compactness and rigidity, thereby enhancing structural stability and catalytic precision [20,23]. Notably, gel filtration analysis indicated that TpXEG12a exists predominantly as a stable dimer in solution, consistent with reports that certain GH12 and GH45 enzymes achieve improved catalytic efficiency and thermostability through dimerization or multimerization [21,24,25]. Dimer formation has been shown to reduce solvent-exposed surface area, strengthen inter-subunit hydrogen bonding networks, and stabilize the active-site architecture, leading to enhanced thermal resistance and substrate affinity [25,26]. Molecular docking supported this hypothesis, suggesting that the dimeric interface of TpXEG12a helps maintain the integrity of the substrate-binding cleft, facilitating more efficient interactions with xyloglucan chains.

In addition, the phylogenetic analysis places TpXEG12a within a fungal GH12 subclade comprising several experimentally characterized xyloglucan-specific enzymes. Despite this clade separation, GH12 xyloglucanases generally share a highly conserved catalytic fold and active-site architecture, indicating that phylogenetic divergence primarily reflects variations in peripheral structural elements rather than fundamental differences in catalytic mechanisms. Sequence diversity among GH12 clades is often concentrated in surface loops, substrate-binding subsites, and regions related to oligomerization, which may influence substrate accessibility, product profiles, and enzymatic properties. Accordingly, the phylogenetic placement of TpXEG12a supports its functional annotation, whereas its high catalytic efficiency and strict substrate specificity are more likely associated with specific structural features rather than clade membership alone.

Compared with other GH12 xyloglucanases from *Aspergillus*, *Penicillium*, *Trichoderma*, and *Rasamsonia* species, TpXEG12a displayed an optimum pH of 4.0 and optimum temperature of 57 °C, categorizing it as a mildly acidic, mesophilic to moderately thermostable fungal GH12 enzyme [5,27,28,29]. Although some GH12s—such as that from *Rasamsonia emersonii*—retain activity at 60–70 °C, TpXEG12a exhibits a notably higher catalytic turnover, suggesting a trade-off between catalytic rate and thermostability within the GH12 family [28]. Moreover, TpXEG12a showed good pH stability (pH 4–8) and moderate thermal tolerance, maintaining nearly full activity after incubation at ≤40 °C for 120 min. These features make TpXEG12a suitable for moderate reaction conditions, although its limited thermostability may restrict direct application under harsh industrial environments.

Another significant advantage of TpXEG12a is its strict substrate specificity for xyloglucan, with almost no detectable cellulolytic activity. Several GH12 xyloglucanases have been reported to exhibit strong substrate preference for xyloglucan with minimal or negligible activity toward cellulose, including enzymes from *Aspergillus niger*, *Aspergillus terreus*, and *Aspergillus oryzae* [5,30,31,32]. In this respect, the substrate specificity of TpXEG12a is consistent with the general functional characteristics of GH12 xyloglucanases, although the selectivity observed in this study appears particularly pronounced (Table S1). This property is critical in industrial applications such as textile biofinishing, paper pulp treatment, and biomass fractionation, where selective xyloglucan hydrolysis without cellulose degradation is essential [33]. MALDI-TOF MS analysis confirmed that TpXEG12a predominantly releases XXXG, XXLG/XLXG, and XLLG oligosaccharides, consistent with a typical endo-acting GH12 xyloglucanase [34,35]. These defined xyloglucan oligosaccharides (XGOs) are also of considerable interest as functional prebiotics, as several studies have shown that XGOs can beneficially modulate gut microbiota and immune responses [36,37].

Beyond its biochemical traits, TpXEG12a demonstrates broad potential across biotechnological applications. In lignocellulosic biomass degradation, xyloglucan acts as a physical cross-linker between cellulose microfibrils, reinforcing cell wall recalcitrance [3]. Selective removal of xyloglucan enhances cellulose accessibility and synergizes with cellulases to improve saccharification efficiency [38]. Accordingly, the co-application of TpXEG12a with cellulases could reduce enzyme loading and process costs, consistent with previous findings on synergistic effects between *Trichoderma reesei* and *Aspergillus aculeatus* xyloglucanases. In the textile industry, the strict specificity of TpXEG12a toward xyloglucan offers an advantage over traditional cellulase-based bio-polishing, minimizing fiber damage and tensile strength loss in cotton and denim fabrics [39,40,41]. Moreover, TpXEG12a may be useful in the feed industry, where controlled degradation of xyloglucan-rich hemicelluloses can improve the digestibility and nutritional quality of soybean meal and cereal-based feeds—a phenomenon previously observed for GH family xyloglucanases [42,43,44].

Despite its high catalytic efficiency, mild reaction requirements, and ideal substrate selectivity, several limitations remain. First, TpXEG12a displays only moderate thermostability compared to thermophilic GH12 enzymes, with rapid activity loss above 50 °C, potentially limiting its high-temperature applicability. Second, the structural conclusions in this study rely primarily on computational modeling and molecular docking; experimental validation using X-ray crystallography or cryo-EM will be required to confirm its dimeric interface and substrate-interaction network. Third, comprehensive validation under simulated industrial conditions—such as biomass pretreatment reactors, textile processing systems, or feed pelleting environments—is still lacking. These aspects warrant further investigation to advance TpXEG12a from laboratory discovery to industrial deployment.

Future research should aim to enhance the thermostability and industrial adaptability of TpXEG12a. Protein engineering strategies, including directed evolution, rational design, and domain fusion, could reinforce the β-sandwich hydrogen-bond network or stabilize the dimer interface to increase thermal tolerance [45,46,47]. Site-directed mutagenesis around the substrate-binding cleft may further optimize substrate affinity and catalytic turnover [48]. Moreover, systematic evaluation of synergistic interactions among TpXEG12a, cellulases, other xyloglucanases, and auxiliary activity (AA) enzymes will be essential to design optimized enzyme cocktails for biomass saccharification. Finally, the enzymatic production of functional xyloglucan oligosaccharides using TpXEG12a could open new opportunities in the food and nutraceutical industries.

In conclusion, TpXEG12a is a highly efficient, structurally distinctive, and substrate-specific GH12 xyloglucanase with broad industrial potential. Its exceptional catalytic efficiency and conserved dimeric configuration provide mechanistic insights and a foundation for future enzyme engineering. Although further optimization is required for certain industrial settings, TpXEG12a significantly enriches the GH12 enzyme repertoire and represents a promising candidate for biomass conversion, textile processing, and functional oligosaccharide production.

## 4. Materials and Methods

### 4.1. Strains, Plasmids, and Culture Conditions

*T. pinophilus* used in this study was obtained from the Microbiology Institute, Jiangxi Academy of Sciences [49]. Strains were routinely cultured on potato dextrose agar (PDA) plates at 30 °C for sporulation. Liquid cultivation for protein expression was conducted in modified Mandels medium supplemented with 2% glucose at 30 °C with shaking at 180 rpm.

Escherichia coli DH5α was used for plasmid construction and maintained in LB medium containing 100 µg/mL ampicillin when required.

All chemicals were of analytical grade. Tamarind xyloglucan (Megazyme, Bray, Ireland), CMC-Na, β-glucan, filter paper (Whatman No. 1, Maidstone, UK), and beechwood xylan were purchased commercially.

### 4.2. Construction of TpXEG12a Overexpression Vector and Strain Transformation

The coding sequence of *TpXEG12a* (without introns) was amplified from *T. pinophilus* genomic DNA using high-fidelity DNA polymerase (Takara, Kusatsu, Japan). The amplified gene was cloned into the fungal expression vector pBIP under the control of the strong constitutive promoter TPcbh1 and fused to a C-terminal His_6_-tag to facilitate purification. The resulting plasmid pBIP-TpXEG12a was linearized with NotI to expose homologous arms for targeted integration at the *cbh1* locus. *T. pinophilus* protoplasts were prepared by digesting young mycelia with 10 mg/mL lysing enzymes from *Trichoderma harzianum* (Sigma-Aldrich, Darmstadt, Germany) for 3–4 h at 30 °C. Approximately 5 μg of linearized pBIP-TpXEG12a DNA was transformed into protoplasts using PEG-CaCl_2_–mediated transformation. Transformants (OEtpXEG12a) were screened on regeneration medium containing 100 µg/mL hygromycin B and verified via PCR and sequencing.

### 4.3. Expression and Purification of Recombinant TpXEG12a

The positive overexpression strain OEtpXEG12a was inoculated into 300 mL Mandels medium and cultured at 30 °C for 96 h. The culture supernatant was collected by centrifugation (10,000× *g*, 10 min, 4 °C), filtered (0.22 µm), and concentrated using a 10 kDa ultrafiltration device. The concentrated supernatant was loaded onto a Ni^2+^–NTA affinity column (GE Healthcare, Marlborough, MA, USA) pre-equilibrated with buffer A (20 mM sodium phosphate, 500 mM NaCl, pH 7.4). After washing with 20 mM imidazole, recombinant TpXEG12a was eluted using 250 mM imidazole. Protein purity was validated by 12% SDS–PAGE, and protein concentration was determined using the Bradford assay. The molecular mass and oligomeric state of TpXEG12a were further analyzed by gel filtration chromatography (Superdex 75 Increase, GE Healthcare) using 20 mM Tris-HCl, 150 mM NaCl, pH 7.5.

### 4.4. Signal Peptide Analysis and N-Terminal Sequencing

Signal peptide prediction was performed using SignalP 6.0 [50], and cleavage sites were predicted based on canonical fungal secretion motifs. Purified mature TpXEG12a was blotted onto a PVDF membrane and subjected to Edman degradation using an automated protein sequencer (Applied Biosystems, Waltham, MA, USA). The first six N-terminal residues were determined and matched to the expected region immediately following the predicted 28-aa signal peptide.

### 4.5. Enzyme Activity Assay

TpXEG12a activity toward tamarind xyloglucan was determined using the DNS method. Standard reactions contained 100 µL of 1% (*w*/*v*) xyloglucan in 50 mM sodium acetate buffer (pH 4.0) and 10 µL diluted enzyme. Mixtures were incubated at optimal temperature for 5 min, followed by termination with 100 µL DNS, boiling for 5 min, cooling, dilution with 800 µL water, and measuring absorbance at 540 nm. One unit of activity (U) was defined as the amount of enzyme releasing 1 µmol of reducing sugars per minute. Assays were performed in triplicate.

### 4.6. Substrate Specificity

Activities toward CMC-Na, β-glucan, filter paper, and beechwood xylan (1% *w*/*v*) were measured under standard assay conditions.

### 4.7. Biochemical Characterization

The optimal reaction temperature of TpXEG12a was determined in 50 mM sodium acetate buffer with pH 4.0 at temperatures ranging from 30 to 90 °C. The thermostability of TpXEG12a was measured by incubating the enzyme solution at different temperatures between 30 and 90 °C for 30 min, 60 min and 120 min in 50 mM sodium acetate buffer with pH 4.0. Then, the residual activities were tested by standard assay.

The optimum pH of TpXEG12a was tested by measuring the enzyme activity under standard assay conditions in different 50 mM buffers between pH 2.0 to 8.0: Glycine-HCl buffer (pH 2.0–3.0), Sodium citrate buffer (pH 3.0–4.0), sodium acetate buffer (pH 4.0–6.0), and Tris-HCl buffer (pH 7.0–8.0). To determine the pH stability of TpXEG12a, the enzyme solution was diluted with different buffers mentioned above and incubated at room temperature (25 °C) for 60 min. Then, the residual activities were tested by standard assay.

Metal ions (1 or 5 mM; added as chlorides) including K^+^, Li^+^, Cu^2+^, Ca^2+^, Mn^2+^, Al^3+^, Zn^2+^, Mg^2+^, Fe^3+^, Ni^+^, and Co^2+^ were added to reaction mixtures; activity without ions was defined as 100%.

### 4.8. Enzyme Kinetics

Xyloglucan solutions at 0.5–10 mg/mL were used to measure initial velocities under optimal conditions. Reducing sugar concentrations were calculated using a glucose standard curve. Km, Vmax, and Kcat values were determined using nonlinear Michaelis–Menten fitting in GraphPad Prism 9.

### 4.9. Analysis of Hydrolysis Products

Hydrolysis reactions (1% xyloglucan, 57 °C, 30 min) were terminated by boiling for 5 min, centrifuged at 12,000× *g*, and the supernatant was mixed with DHB matrix for MALDI-TOF MS analysis. Mass spectra were acquired using a Bruker Autoflex speed MALDI-TOF/TOF instrument (Billerica, MA, USA) in positive-ion reflector mode. Characteristic oligosaccharides (XXXG, XXLG/XLXG, XLLG) were identified based on *m*/*z* values.

### 4.10. Structural Prediction and Molecular Docking

The 3D structure of TpXEG12a was predicted using AlphaFold 3 [51], and structural visualization was performed using PyMOL 2.5. Docking between TpXEG12a and xyloglucan fragments was performed using AutoDock Vina 1.2. The three-dimensional structure of the xyloglucan substrate used for molecular docking was constructed based on a representative repeating unit of tamarind xyloglucan. The modeled substrate consists of a β-(1 → 4)-linked D-glucan backbone substituted with α-D-xylopyranosyl residues at the O-6 position of selected glucose units, corresponding to the commonly observed XXXG-type motif. The initial xyloglucan structure was generated using standard carbohydrate geometries and subsequently energy-minimized prior to docking. This oligomeric xyloglucan fragment was employed as the ligand in the docking simulations to represent the binding behavior of TpXEG12a toward native xyloglucan substrates.

### 4.11. Phylogenetic Analysis

The amino acid sequence of TpXEG12a and 14 homologous GH12 xyloglucanases from fungi were aligned using Clustal Omega 1.2. Phylogenetic trees were constructed in MEGA 11.0 using the Neighbor-Joining method with 1000 bootstrap replicates. Evolutionary distances were computed using the p-distance method, and sites with <50% coverage were removed.

### 4.12. Synergistic Degradation with Cellulase

Soybean meal, corncob powder, and cotton fibers were incubated with (i) buffer, (ii) TpXEG12a (10 U), (iii) commercial cellulase (10 FPU), or (iv) TpXEG12a + cellulase. Reactions were performed at 50 °C for 12 h in 50 mM sodium acetate (pH 4.8). Released glucose was quantified using DNS. Synergy was defined when combined activity exceeded additive effects.

## Data Availability

Additional information regarding the data can be obtained by contacting the corresponding authors.

## Supplementary Materials

The following supporting information can be downloaded at: https://www.mdpi.com/article/10.3390/ijms27010294/s1. Reference [52] is cited in the supplementary materials.

## Abbreviations

The following abbreviations are used in this manuscript:

XEG	xyloglucanase
GH12	glycoside hydrolase family 12
SEC	size-exclusion chromatography
CMC	carboxymethyl cellulose
XGO	xyloglucan oligosaccharides
