# Supplementary material for: Characterization of Xyloglucanase TpXEG12a from Talaromyces pinophilus
Source: Int J Mol Sci. 2025 Dec 27;27(1):294. doi: 10.3390/ijms27010294 (PMC12786156; doi:10.3390/ijms27010294)
Supplement: Supplementary file 1 [file ijms-27-00294-s001.zip › ijms-4038087-supplementary.pdf]

Table S1. Comparison of enzymatic properties of TpXEG12a and representative GH12 xyloglucanases reported in the literature

Enzyme	Source organism	GH family	Optimum pH	Optimum temperature (°C)	Km (mg mL⁻¹)	kcat (s⁻¹)	kcat/Km (s⁻¹·mg⁻¹·mL)	Vmax / Specific activity	Substrate specificity	Reference
TpXEG12a	Talaromyces pinophilus	GH12	See Results	See Results	3.715	922.69	248.37	2375 U mg⁻¹	Highly specific to xyloglucan; negligible activity toward cellulose	This study
AnXEG12A	Aspergillus niger	GH12	5.0	50–60	0.54	49	~90.7	113 μmol min⁻¹ mg⁻¹	Strictly xyloglucan-specific	[5, 52]
XegA	Aspergillus niveus	GH12	5.5	60	1.50	144.31	~96.21	—	Xyloglucan-specific	[29]
AtXEG12	Aspergillus terreus	GH12	5.5	65	1.20	—	—	17.4 μmol min⁻¹ mg⁻¹	Xyloglucan-specific	[28]
Xeg12A	Aspergillus oryzae	GH12	~5.5	~60	—	—	—	—	Produces XXXG, XLXG, XXLG, and XLLG	[27]

Notes: Kinetic parameters were determined using different xyloglucan substrates (e.g., tamarind seed, pea, or citrus xyloglucan) and under varying assay conditions (pH, temperature, enzyme expression systems). Therefore, direct quantitative comparisons across studies should be interpreted with caution. Where kcat/Km values were not explicitly reported, they were calculated from published Km and kcat values when available. Units and symbols are formatted according to IJMS guidelines.
